# Natural Exposure of Horses to Mosquito-Borne Flaviviruses in South-East Queensland, Australia

**DOI:** 10.3390/ijerph10094432

**Published:** 2013-09-17

**Authors:** Natalie A. Prow, Cindy S. E. Tan, Wenqi Wang, Jody Hobson-Peters, Lisa Kidd, Anita Barton, John Wright, Roy A. Hall, Helle Bielefeldt-Ohmann

**Affiliations:** 1 Australian Infectious Diseases Research Centre, University of Queensland, St. Lucia, QLD 4078, Australia; E-Mails: n.prow@uq.edu.au (N.A.P.); s.tan5@uq.edu.au (C.S.E.T.); j.peters2@uq.edu.au (J.H.-P.); r.hall@uq.edu.au (R.A.H.); 2 School of Biochemistry & Molecular Biosciences, University of Queensland, Brisbane, QLD 4072, Australia; 3 School of Veterinary Science, University of Queensland, Gatton, QLD 4343, Australia; E-Mails: w.wang3@uq.edu.au (W.W.); l.kidd@uq.edu.au (L.K.); a.scampton@uq.edu.au (A.B.); a.scampton@uq.edu.au (J.W.)

**Keywords:** flavivirus, equine, antibody response, mosquito-borne, encephalitis

## Abstract

In 2011 an unprecedented epidemic of equine encephalitis occurred in south-eastern (SE) Australia following heavy rainfall and severe flooding in the preceding 2–4 months. Less than 6% of the documented cases occurred in Queensland, prompting the question of pre-existing immunity in Queensland horses. A small-scale serological survey was conducted on horses residing in one of the severely flood-affected areas of SE-Queensland. Using a flavivirus-specific blocking-ELISA we found that 63% (39/62) of horses older than 3 years were positive for flavivirus antibodies, and of these 18% (7/38) had neutralizing antibodies to Murray Valley encephalitis virus (MVEV), Kunjin virus (WNV_KUN)_ and/or Alfuy virus (ALFV). The remainder had serum-neutralizing antibodies to viruses in the Kokobera virus (KOKV) complex or antibodies to unknown/untested flaviviruses. Amongst eight yearlings one presented with clinical MVEV-encephalomyelitis, while another, clinically normal, had MVEV-neutralizing antibodies. The remaining six yearlings were flavivirus antibody negative. Of 19 foals born between August and November 2011 all were flavivirus antibody negative in January 2012. This suggests that horses in the area acquire over time active immunity to a range of flaviviruses. Nevertheless, the relatively infrequent seropositivity to MVEV, WNV_KUN_ and ALFV (15%) suggests that factors other than pre-existing immunity may have contributed to the low incidence of arboviral disease in SE-Queensland horses during the 2011 epidemic.

## 1. Introduction

More than 70 arthropod-borne viruses (“arboviruses”) have been isolated in Australia, but only a few have been implicated in human and livestock diseases [1]. The family *Flaviviridae* comprises three genera, the flaviviruses, pestiviruses and “hepatitis C-like viruses”. Only the flavivirus genus contains viruses that are transmitted by arthropods. Flaviviruses are small, 40–55 nm in diameter, enveloped, single-stranded, positive sense RNA-viruses with a genome of ~11 kb which encodes three structural and seven non-structural proteins. The flaviviruses have been placed in nine serologically defined groups, with an additional few still to be classified. The type species of the flavivirus genus is yellow fever virus, with other major members being the dengue viruses, Japanese encephalitis virus (JEV) and the tick-borne encephalitis viruses [1]. Flaviviruses cause a wide variety of symptoms in humans and other mammals ranging from subclinical infection to mild fever, fatigue, myalgia, arthralgia, severe hemorrhagic syndromes or hepatitis and meningo-encephalitis [2].

In Australia, Murray Valley encephalitis virus (MVEV) and a strain of West Nile virus (WNV) named Kunjin virus (WNV_KUN_) are the main aetiologic agents of viral encephalitis in humans, occurring as annual isolated cases or sporadic outbreaks, mainly in the north-western part of the continent [1]. MVEV is the more virulent pathogen, with morbidity and case fatality rates of 0.1–1.0% and 15–20%, respectively, and the only proven cause of fatal human cases [1]. In contrast, WNV_KUN_ infections are less frequent and less severe [3]. Horses have been described as a susceptible species and have been involved in WNV outbreaks overseas, most notably the outbreak that commenced in the USA in 1999 [4]. In Australia, infection with WNV_KUN_ has been observed in horses, but the disease has rarely been confirmed [5], while cases of equine MVEV-encephalitis are mostly, but not exclusively, seen under the same environmental conditions as those precipitating human MVEV cases (Barton *et al.*, [6], unpublished data). Other flaviviruses circulating in Australia, but generally considered apathogenic or with very low virulence in mammals include Kokobera virus (KOKV), Stratford virus (STRV), Alfuy virus (ALFV) and Edge Hill virus (EHV) [7,8,9,10]. An incursion of JEV occurred in 1995 in the Torres Strait, but the virus did not become endemic and has not been diagnosed on the Australian mainland since 1998 [11,12,13].

Outbreaks of MVEV and WNV_KUN_ encephalitis generally occur when heavy rainfall in northern and central Australia facilitates southward movement of the virus via infected water birds to flooded wetlands in south-east of the continent, notably the Murray Valley. These flooded areas provide an ideal environment for proliferation of mosquitoes which facilitates spillover from amplifying hosts (waterfowl) to susceptible hosts (horses and humans). Indeed, this scenario occurred in early 2011 following extensive flooding in the Murray-Darling basin and some other inland river systems with an unprecedented outbreak of equine encephalitis in horses in south-eastern Australia [14,15]. There were 982 reported cases of horses displaying clinical signs consistent with arboviral disease with a case fatality rate of 5–20% [15]. Isolation and sequencing of a virus from the brains of fatal cases in New South Wales (NSW) revealed the aetiological agent for many cases to be a variant strain of WNV, most closely related to WNV_KUN_ and named WNV_NSW2011_ [14]. Other fatal cases were due to MVEV. Despite very extensive flooding throughout Eastern Queensland, less than 6% of the reported cases of arboviral disease came from this state and of these less than half were diagnosed as caused by either WNV_KUN_ or MVEV [15]. This prompted us to conduct a small-scale survey of the equine population at the University of Queensland (UQ) Gatton Campus, located in the Lockyer Valley, approximately 80 km west of Brisbane, and one of the more severely flood-affected areas during early 2011, with the aim of assessing flavivirus activity that might confer immunity and protection against flavivirus encephalitis.

## 2. Experimental Section

### 2.1. Animals

Blood samples were obtained from a total of 95 horses from the University of Queensland teaching and research equine herds, including 25 foals (0 to 5 months), eight yearlings and 62 adult horses (3–35 years). The majority of the horses were standardbred, thoroughbred or stock horse-standardbred crosses. All but six horses were either born at the Gatton Campus (all foals and yearlings and most of the adult thoroughbred horses) or had been pastured there for a minimum of 4 years, and most of the latter group originated from Queensland. The study was approved by the University of Queensland Animal Ethics Committee (approval numbers SVS/306/11/VAXINE and ANRFA/318/12).

### 2.2. Sample Collection

Blood samples were collected by venepuncture into vacutainers (BD Biosciences, Franklin Lakes, NJ, USA) and allowed to clot at room temperature, then centrifuged at 4 °C for 10 min at 3,500 rpm. Procured serum was stored at −20 °C in sterile cryovials until assayed by ELISA and virus neutralization following heat inactivation at 56 °C for 30 min.

### 2.3. Flavivirus Blocking ELISA

All horse sera were originally screened for flavivirus-specific antibodies using a modification of an epitope-blocking ELISA [16,17]. U-bottomed 96-well polyvinyl chloride plates (Costar, Corning, NY, USA) were coated with an optimal concentration of flavivirus antigen [17] diluted in coating buffer (0.1 M carbonate/bicarbonate, pH 9.6) at 50 µL/well by incubation overnight at 4 °C. The plates were then washed twice in wash buffer (PBS with 0.05% Tween-20), and nonspecific sites were blocked with 100 µl blocking buffer (0.05 M Tris, 1 mM EDTA, 0.15 M NaCl, 0.05% (v/v) Tween-20, 0.2% (w/v) casein, pH 8.0) for 1 h at room temperature (RT). Reference or test sera were added (50 µL/well) in duplicate at dilutions of 1/5 and 1/10 in blocking buffer and incubated for 1 h at RT. Non-immune horse sera were used as negative controls. Without removal of serum, 50 μL of pan-reactive monoclonal antibody (Mab) 4G2, targeting the flavivirus envelope protein [18,19], was added to each well and after gentle agitation the plates were incubated at RT for 1 h. Plates were washed four times and bound Mab detected by incubation for 1 h at RT with horseradish peroxidase-conjugated goat anti-mouse immunoglobulin pre-adsorbed for equine serum proteins (Jackson ImmunoResearch Laboratories, West Grove, PA, USA) diluted in blocking buffer. Plates were washed six times and enzyme activity visualised by the addition of 100 µL substrate solution 1 mM (2,2′-azino-bis(3-ethylbenzthiazolinesulfonic acid); ABTS) and 3 mM H_2_O_2_ in a citrate/phosphate buffer, pH 4.2. Quantitative results were determined by measuring the optical density (OD) at 405 nm, and percent inhibitions were calculated at 100—(OD (test)/OD (negative control) × 100). The cut-off for positivity was ≥30% inhibition and only positive samples were tested in the virus neutralization assays [16,17]. A similar blocking ELISA format was used to test for MVEV- and KUNV-specific antibodies using the Mab 10C6 [18] and 3.1112G [17], respectively.

### 2.4. Virus Neutralization Assay

Heat-inactivated test sera were titrated in doubling dilutions from 1:20 to 1:2,560 in Dulbecco’s modified Eagle’s medium (DMEM) without fetal bovine serum (FBS) in wells of a 96-well microtitre plate (Costar, Corning, NY, USA). Approximately 100 infectious units of MVEV (strain 1–51), WNV_KUN_ (strain MRM16), ALFV (strain MRM3929), STRV (strain C338) or KOKV (strain MRM32) diluted in DMEM with 2% FBS were added to each well (50 µL) containing diluted serum (50 µL) and plates were incubated at 37 °C for an hour. Vero or porcine-stable equine kidney (PSEK) cells [20], resuspended in 50 µL of medium, were then added to each well at a density of 2 × 10^5^ cells/well and the plates were incubated for 5 days at 37 °C in a humidified CO_2_-incubator before microscopic examination for cytopathic effect (CPE). Cells were also added to a 1:20 dilution of each serum in the absence of virus to ensure that the samples were not toxic to the cells. The neutralisation titre was expressed as the reciprocal of the highest serum dilution where CPE was recorded. The cells were fixed *in situ* in the plates and subjected to an ELISA protocol [21] using anti-flaviviral E protein antibody, Mab 4G2, and pre-absorbed secondary antibody-conjugate to ascertain viral replication.

## 3. Results and Discussion

Of 62 adult horses (age range 3–35 years) and eight yearlings tested 41 were positive in the flavivirus-specific blocking ELISA (63%), and of these 31 had neutralizing antibodies to one or more of the following flaviviruses: MVEV, KUNV, ALFV, STRV and KOKV (Table 1 and Table 2). Blocking ELISAs using antibodies specific for MVEV (10C6) and KUNV (3.1112G) were also used to further analyse positive serum samples in the flavivirus-specific blocking ELISA. Horses positive for MVEV by blocking ELISA also had neutralizing antibodies against MVEV (T141, T143). However, some horses positive in the KUN blocking ELISA did not have detectable neutralizing antibodies against KUNV (T131, R10, T105 and T129). Seven horses were judged to have either given a false positive reaction in ELISA or, more likely, were positive for an unknown/untested flavivirus, such as EHV. Of the eight yearlings, all of whom were born at the UQ Gatton Campus, two had neutralizing antibodies to both MVEV and KOKV. One of the MVEV-positive yearlings (T143) presented with clinical encephalomyelitis and was eventually euthanatized due to persistent neurological sequela (Barton *et al.*, unpublished data). The one MVEV-positive yearling not showing symptoms (T141) was retested again in 2013 at age 3 years and was found to be strongly positive in the 4G2 blocking ELISA and had retained the same level of MVEV-neutralizing antibodies (data not shown). 

A total of 19 foals were born in the UQ herd between August and November of 2011, 11 by flavivirus antibody negative mares and eight to mares with KOKV-specific antibodies. The foals were serologically tested in January 2012, at which time they were between 74 and 152 days old. All 19 foals were negative in the flavivirus blocking-ELISA (included in the ≤2 years group in Table 1). Another six foals born in 2012 were all negative at birth, but two received passive antibody transfer via colostrums from naturally infected mares, and were still positive in the blocking ELISA 2–2.5 months after birth (data not shown). Two foals, one of which had received passive antibody transfer, were lost due to severe injuries. The remaining four foals were retested at the age of 6–7 months and all were still negative in the blocking ELISA.

**Table 1:** Age distribution of horses from which sera was collected and assessed for antibodies to flaviviruses in blocking ELISA.

Age Group (years) *	Total Tested	No. of samples positive in flavi blocking ELISA ^#^	Percentage of horses positive in flavi blocking ELISA
≤ 2	33	2	6
3–10	27	13	48
11–20	29	21	72
21–35	6	5	83
Total	95	41	43

* Age at time of serum collection in 2011; ^#^ Blocking ELISA inhibition values ≥30 are considered significant.

Ten horses (eight in the 11–20 years group and two in the >21 years group) had been employed in a JEV-vaccine trial during 2008 [22], and all were positive in the 4G2-flavivirus blocking ELISA. However, only three of these had neutralizing antibodies to MVEV (T106, T116, T115; Table 2). Since these three horses also had neutralizing antibody titres to ALFV equal to or higher than the MVEV neutralizing titre, it is more likely that the antibodies were induced by either or both of these viruses in the years since the vaccination rather than being cross-reactivity induced by vaccination with inactivated JEV. This supposition is further supported by the fact that MVEV-infection occurred in two yearlings in this location in 2011, and all ten horses still had low neutralizing antibody titres to JEV, yet seven were negative for MVEV, KUNV and ALFV. 

Neutralizing antibodies to KOKV was by far the most common source of flavivirus reactivity, with 25 of 40 flavivirus antibody positive horses having neutralizing antibodies to this virus (70% of flavivirus-positive horses; Table 2). 

**Table 2:** Serological analysis of sera that were positive in the Flavi-blocking ELISA ^†^.

Sample ID ^†^	Age *	Blocking ELISA ^#^			Virus Neutralization Assay Titres					
		Flavi (4G2)	MVEV (10C6)	KUNV (3.1112G)	MVEV	KUNV	ALFV	STRV	KOKV	Neutralization Diagnosis^₸^
R1	5	+	−	−	−	−	−	−	20	KOKV
R10	10	+	+	+	−	−	ND	ND	ND	unknown
R15	5	+	−	−	−	−	−	−	−	unknown
R21	12	+	−	−	−	−	−	−	80	KOKV
R26	12	+	−	−	−	−	−	−	40	KOKV
R41	4	+	−	−	−	−	−	−	40	KOKV
R44	10	+	−	−	−	−	−	−	80	KOKV
R5	9	+	−	−	−	−	−	−	20	KOKV
R7	5	+	−	−	−	−	20	−	−	ALFV
T101	14	+	−	+	−	80	−	−	40	KUNV/KOKV
T103 ^Ɣ^	11	+	−	−	−	−	−	−	40	KOKV
T104	18	+	−	−	−	−	−	80	40	STRATV/KOKV
T105	15	+	−	+	−	−	−	80	−	STRATV
T106 ^Ɣ^	≥8	+	−	−	20	−	160	80	40	ALFV/STRATV
T107	15	+	−	−	−	−	−	−	20	KOKV
T108 ^Ɣ^	35	+	−	−	−	−	−	−	40	KOKV
T109	22	+	−	−	−	−	−	−	−	unknown
T111	19	+	−	−	−	−	−	80	80	STRATV/KOKV
T112	15	+	−	−	−	20	−	−	40	KUNV/KOKV
T113^Ɣ^	10	+	−	−	−	−	−	80	20	STRATV
T114 ^Ɣ^	15	+	+	−	−	−	−	80	−	STRATV
T115 ^Ɣ^	18	+	+	+	80	−	40	160	20	STRATV/MVEV
T116 ^Ɣ^	15	+	+	−	40	40	40	160	40	STRATV
T118 ^Ɣ^	19	+	+	+	−	−	−	80	−	STRATV
T119 ^Ɣ^	15	+	−	−	−	−	−	160	20	STRATV
T122	9	+	−	−	−	−	−	−	−	unknown
T124	26	+	−	−	−	−	−	160	−	STRATV
T125	19	+	−	−	−	−	40	40	20	STRATV/ALFV/KOKV
T127 ^Ɣ^	14	+	+	−	−	−	−	−	20	KOKV
T129	21	+	−	+	−	−	−	−	−	unknown
T131	≥8	+	−	+	−	−	−	−	−	unknown
T135	12	+	−	−	−	−	80	−	80	ALFV/KOKV
T141	1	+	+	−	20	−	20	−	40	MVEV/ALFV/KOKV
T142	21	+	−	−	−	−	−	−	20	KOKV
T143	1	+	+	−	160	−	−	−	20	MVEV
T145	7	+	−	−	−	−	−	−	−	unknown
T146	4	+	−	−	−	−	−	−	40	KOKV

* Approximate age at the time of serum collection in 2011; ^#^ Blocking ELISA inhibition values ≥30 are considered positive, “**+**” positive; “**−**” negative; ^₸^ Virus neutralization titres that differ by ≥ 4 fold are considered significant; ^Ɣ^ Horses were part of a JEV vaccine trial in 2008 [21]; ^†^ The sera from 4 horses that were positive by the flavi blocking ELISA, but were not assessed in any of the other assays have not been included in this table; ND—not done; MVEV—Murray Valley encephaltitis virus; KUNV—West Nile virus (Kunjin virus); ALFV—Alfuy virus; STRATV—Stratford virus; KOKV—Kokobera virus.

A previous serological study in 2008 of 13 horses (10 of which are also included in the 2011 cohort) demonstrated KOKV specific neutralizing antibodies in five horses (38%; [10]). Of these five only four horses were available for inclusion in the present study and only three of these horses still had antibodies to KOKV in 2011, although in one case this might have been cross reactivity to STRATV, suggesting that exposure to the virus is stochastic and possibly relatively infrequent. The natural host for KOKV is unknown, but wallabies and kangaroos have been surmised [23]. Furthermore, horses have also been suggested to contribute to maintenance of KOKV [10,23]. Also, large numbers of potential avian hosts (e.g., egrets (*Ardeola ibis*, *Egretta* spp.), herons (*Nycticorax caledonicus*), ibises (*Threskiornis molucca*, *T. spinicollis*), magpie geese (*Anseranas semipalmata*), whistling ducks (*Dendrocygna eytoni*), swamphens (*Porphyrio porphyrio*) and others) are associated with wetlands on the campus and surrounding areas, and an abundance of mosquitoes, notably *Culex annulirostris*, the main vector for the viruses included in this study ([24,25]; Andrew van den Hurk, personal communication, 2013). Antibody levels may decline relatively rapidly in the absence of re-exposure. This is in agreement with other studies showing that natural flavivirus exposure furnishes only relatively transient immunity [26], but contrast with studies of yellow fever virus-vaccination in humans in whom persistence of neutralizing antibodies for up to 30–35 years was demonstrated [27]. Possible explanations would be animal species and/or virus strain differences, infection dose and local *versus* systemic replication of virus [26,28,29,30].

It has been suggested that equines might be useful sentinels for flavivirus activity [31,32,33,34,35]. Thus, Nielsen *et al.* [32] concluded that immunologically naive horses are more sensitive for detection of WNV transmission than mosquito sampling alone. Similarly, Phoutrides *et al.* [34] concluded that horses constitute an important sentinel species for WNV in regions where they may have greater geographical range than other potential sentinel species. This is in contrast to Patnaik *et al.* [31] who found that birds, followed by mosquitoes and equines best predicted the occurrence of human WNV-disease cases in an urban area, while sentinel chickens had extremely low predictive success. The prerequisite for the usefulness of equines as sentinels would, of course, be absence of general use of JEV- and/or WNV-vaccination. Since in Australia JEV-vaccination is generally only used for horses being exported or going for competition in South-East Asia, and no WNV-vaccine is yet licensed and in use in Australia, using equines as sentinels for flavivirus activity could be given consideration, notably in the face of the 2011 WNV/MVEV epidemic in SE-Australia [15]. Our data, while based on a geographically limited survey, suggest that exposure to flaviviruses is a relatively infrequent event, at least in the study area, but that the likelihood of it occurring increases with the age of the animal, albeit at a slow rate. This is further supported by preliminary data from a 2012–13 survey of 118 horses, aged 2.5–7 years, racing at several venues in SEQ; less than 6% were positive in the flavivirus-blocking ELISA, and only half of these had low neutralizing antibody titres to WNV_KUN_, while none had antibodies to MVEV (unpublished data). It therefore seems advisable to focus on young horses less than 10 years as sentinels. Since viruses of the KOKV complex do not induce cross-neutralizing antibodies to MVEV and WNV_KUN_ [10] and in our study only 10 out of 95 horses (10.5%) had neutralizing antibodies to MVEV, KUNV, or ALFV it seems unlikely that pre-existing immunity is the sole explanation for the limited occurrence of equine flavivirus encephalitis in QLD during the 2011 equine epidemic. This conclusion is further supported by the data from the race-horses. Other factors to consider would be ecological factors such as bird movements and a “flushing effect” due to the severity of rainfall and flooding [32,36], or the presence of a cryptic host, e.g., rabbits, another bird species or another mammal, in the affected areas of SE-Australia. Variation in vector competence of different mosquito populations could also be considered. While *Culex annulirostris* is the major vector involved in transmission of flaviviruses in Australia and all isolates of WNV_NSW2011_ isolated from field-caught mosquitoes were from this species [14], then a previous study by Jansen *et al.* [37] demonstrated intraspecies variation in vector competence between populations. Notably, *C. annulirostris* gave an overall high level of vector competence for WNVNY99, but mosquitoes collected from Brisbane (SEQ) appeared less efficient vectors compared to populations collected from Sydney (NSW) and Cairns (North QLD). Thus, intraspecies variations of the main vector may have contributed to differences seen in transmission in different areas during the outbreak and may, at least in part, explain the low incidence of arboviral disease in SEQ.

## 4. Conclusions

While a wide range of mosquito-borne flaviviruses circulate in SE-Queensland and horses in the region over time may acquire immunity to these viruses, the current survey suggests that horses under the age of 10 years could function as sentinels for medically important flaviviruses including WNV, MVEV and JEV. Moreover, further studies into the epidemiology and dynamics of WNV_KUN,_ in particular, are warranted, as pre-existing immunity to flaviviruses of the JEV sero-complex clearly is not the explanation for the low incidence of arboviral disease in SE-Queensland horses during the 2011 epidemic.

## References

[1] Mackenzie J.S., Hall R.A., Lindsay M.D., Bielefeldt-Ohmann H., Poidinger M., Thompson R.C.A. (2000). Molecular Epidemiology and Phylogeny of Australian Arboviruses. Molecular Epidemiology of Infectious Diseases.

[2] Gubler D., Kuno G., Markoff L., Knipe D.M., Howley P.M. (2006). Flaviviruses. Field’s Virology.

[3] Hall R.A., Broom A.K., Smith D.W., Mackenzie J.S. (2002). The ecology and epidemiology of Kunjin virus. Curr. Top. Microbiol. Immunol..

[4] Garmendia A.E., Van Kruiningen H.J., French R.A. (2001). The West Nile virus: Its recent emergence in North America. Microbes Infect..

[5] Studdert M.J. (2003). West Nile virus revisited and other mosquito borne viruses of horses in Australia. Aust. Vet. J..

[6] Gordon A.N., Marbach C.R., Oakey J., Edmunds G., Condon K., Diviney S.M., Williams D.T., Bingham J. (2012). Confirmed case of encephalitis caused by Murray Valley encephalitis virus infection in a horse. J. Vet. Diagn. Invest..

[7] Poidinger M., Hall R.A., Lindsay M.D., Broom A.K., Mackenzie J.S. (2000). The molecular epidemiology of Kokobera virus. Virus Res..

[8] Nisbet D.J., Lee K.J., van den Hurk A.F., Johansen C.A., Kuno G., Chang G.J., Mackenzie J.S., Ritchie S.A., Hall R.A. (2005). Identification of new flaviviruses in the Kokobera virus complex. J. Gen. Virol..

[9] Macdonald J., Poidinger M., Mackenzie J.S., Russell R.C., Doggett S., Broom A.K., Phillips D., Potamski J., Gard G., Whelan P. (2010). Molecular phylogeny of Edge Hill virus supports its position in the yellow fever virus group and identifies a new genetic variant. Evol. Bioinform. Online.

[10] May F.J., Clark D.C., Pham K., Diviney S.M., Williams D.T., Field E.J., Kuno G., Chang G.J., Cheah W.Y., Setoh Y.X. (2013). Genetic divergence among members of the Kokobera group of flaviviruses supports their separation into distinct species. J. Gen. Virol..

[11] Hanna J.N., Ritchie S.A., Phillips D.A., Shield J., Bailey M.C., Mackenzie J.S., Poidinger M., McCall B.J., Mills P.S. (1996). An outbreak of Japanese encephalitis in the Torres Strait, Australia, 1995. Med. J. Aust..

[12] Hanna J.N., Ritchie S.A., Phillips D.A., Lee J.M., Hills S.L., van den Hurk A.F., Pyke A.T., Johansen C.A., Mackenzie J.S. (1999). Japanese encephalitis in north Queensland, Australia, 1998. Med. J. Aust..

[13] Van den Hurk A.F., Montgomery B.L., Northill J.A., Smith I.L., Zborowski P., Richie S.A., Mackenzie J.S., Smith G.A. (2006). Short report: The first isolation of Japanese encephalitis virus from mosquitoes collected from mainland Australia. Am. J. Trop. Med. Hyg..

[14] Frost M.J., Zhang J., Edmonds J.H., Prow N.A., Gu X., Davis R., Hornitzky C., Arzey K.E., Finlaison D., Hick P. (2012). Characterization of virulent West Nile virus Kunjin strain, Australia, 2011. Emerg. Infect. Dis..

[15] Roche S.E., Wicks R., Garner M.G., East I.J., Paskin R., Moloney B.J., Carr M., Kirkland P. (2013). Descriptive overview of the 2011 epidemic of arboviral disease in horses in Australia. Aust. Vet. J..

[16] Hall R.A., Broom A.K., Hartnett A.C., Howard M.J., Mackenzie J.S. (1995). Immunodominant epitopes on the NS1 protein of MVE and KUN viruses serve as targets for a blocking ELISA to detect virus-specific antibodies in sentinel animal serum. J. Virol. Methods.

[17] Blitvitch B.J., Bowen R.A., Marlenee N.L., Hall R.A., Bunning M.L., Beaty B.J. (2003). Epitope-blocking enzyme-linked immunosorbent assays for detection of West Nile virus antibodies in domestic mammals. J. Clin. Microbiol..

[18] Broom A.K., Hall R.A., Johansen C.A., Oliveira N., Howard M.A., Lindsay M.D., Kay B.H., Mackenzie J.S. (1998). Identification of Australian arboviruses in inoculated cell cultures using monoclonal antibodies in ELISA. Pathology.

[19] Gentry M.K., Henchal E.A., McCown J.M., Brandt W.E., Dalrymple J.M. (1982). Identification of distinct antigenic determinants on dengue-2 virus using monoclonal antibodies. Am. J. Trop. Med. Hyg..

[20] Gorman B.M., Leer J.R., Filippich C., Goss P.D., Doherty D.L. (1975). Plaquing and neutralization of arboviruses in the PS-EK line of cells. Aust. J. Med. Technol..

[21] Hobson-Peters J., Toye P., Sánchez M.D., Bossart K.N., Wang L.F., Clark D.C., Cheah W.Y., Hall R.A. (2008). A glycosylated peptide in the West Nile virus envelope protein is immunogenic during equine infection. J. Gen. Virol..

[22] Lobigs M., Pavy M., Hall R.A., Lobigs P., Cooper P., Komiya T., Toriniwa H., Petrovsky N. (2010). An inactivated Vero cell-grown Japanese encephalitis vaccine formulated with Advax, a novel inulin-based adjuvant, induces protective neutralizing antibody against homologous and heterologous flaviviruses. J. Gen. Virol..

[23] Doherty R.L., Carley J.G., Gorman B.M. (1964). Studies of arthropod-borne virus infections in Queensland. IV. Further serological investigations of antibodies to group B arboviruses in man and animals. Aust. J. Exp. Bio. Med. Sci..

[24] Russell R.C. (1998). Vector *vs.* humans in Australia—Who is on top down under? An update on vector-borne disease and research on vectors in Australia. J. Vector Ecol..

[25] Russell R.C., Kay B.H. (2004). Medical entolomology: Changes in the spectrum of mosquito-borne disease in Australia and other vector threats and risks, 1972–2004. Aust. J. Entomol..

[26] Shirafuji H., Kanehira K., Kamio T., Kubo T., Shibahara T., Konishi M., Murakami K., Nakamura Y., Yamanaka T., Kondo T. (2009). Antibody responses induced by experimental West Nile virus infection with or without previous immunization with inactivated Japanese encephalitis vaccine in horses. J. Vet. Med. Sci..

[27] Poland J.D., Calisher C.H., Monath T.P., Downs W.G., Murphy K. (1981). Persistence of neutralizing antibody 30–35 years after immunization with 17D yellow fever vaccine. Bull. WHO..

[28] Reisen W.K., Chiles R.E., Kramer L.D., Martinez V.M., Eldridge B.F. (2000). Method of infection does not alter response of chicks and house finches to western equine encephalomyelitis and St. Louis encephalitis viruses. J. Med. Entomol..

[29] Konishi E., Shoda M., Kondo T. (2006). Analysis of yearly changes in levels of antibodies to Japanese encephalitis virus nonstructural 1 protein in racehorses in central Japan shows high levels of natural virus activity still exist. Vaccine.

[30] Martín J., Hermida L., Castro J., Romero Y., Cardosa J., Guillén G. (2009). Viremia and the magnitude of the immune response upon infection of green monkeys with dengue virus type 2 are strain-dependent. Curr. Microbiol..

[31] Patnaik J., Juliusson L., Vogt R.L. (2007). Environmental predictors of human West Nile virus infections, Colorado. Emerg. Infect. Dis..

[32] Nielsen C.F., Reisen W.K., Armijos M.V., MacLachlan N.J., Scott T.W. (2008). High subclinical West Nile virus incidence among nonvaccinated horses in Northern California associated with low vector abundance and infection. Am. J. Trop. Med. Hyg..

[33] Ward M.P., Scheurmann J.A. (2008). The relationship between equine and human West Nile virus disease occurrence. Vet. Microbiol..

[34] Phoutrides E., Jusino-Mendez T., Perez-Medina T., Seda-Lozada R., Garcia-Negron M., Davila-Toro F., Hunsperger E. (2011). The utility of animal surveillance in the detection of West Nile virus activity in Puerto Rico, 2007. Vector Borne Zoonotic Dis..

[35] Rushton J.O., Lecollinet S., Hubálek Z., Svobodová P., Lussy H., Nowotny N. (2013). Tick-borne encephalitis virus in horses, Austria, 2011. Emerg. Infect. Dis..

[36] Doggett S.L., Russell R.C., Clancy J., Haniotis J., Cloonan M.J. (1999). Barmah Forest virus epidemic on the south coast of New South Wales, Australia, 1994–1995: Viruses, vectors, human cases, and environmental factors. J. Med. Entomol..

[37] Janssen C.C., Webb C.E., Northill J.A., Ritchie S.A., Russell R.C., van den Hurk A.F. (2008). Vector competence of Australian mosquito species for a North American strain of West Nile virus. Vector Borne Zoonotic Dis..

